# Editorial: Interplay between gut microbiota and the immune system in liver surgery and liver diseases

**DOI:** 10.3389/fcimb.2023.1188092

**Published:** 2023-03-27

**Authors:** Julio Plaza-Díaz, Luis Fontana, Ana I. Álvarez-Mercado

**Affiliations:** ^1^ Instituto de Investigación Biosanitaria ibs.GRANADA, Complejo Hospitalario Universitario de Granada, Granada, Spain; ^2^ Department of Biochemistry and Molecular Biology II, School of Pharmacy, University of Granada, Granada, Spain; ^3^ Children's Hospital of Eastern Ontario Research Institute, Ottawa, ON, Canada; ^4^ Institute of Nutrition and Food Technology, Biomedical Research Center, University of Granada, Granada, Spain

**Keywords:** microbiota, microbiome, liver diseases, liver surgery, gut-liver axis

## Gut microbiota and liver diseases

Microbiota refers to the assemblage of microbes present in the environment (as first defined by Lederberg and McCray (Lederberg and Mccray, 2001). There is no conclusive evidence that individuals, or even different body locations, harbor a “core” set of these microorganisms. In this regard, every person harbors a symbiotic community of microbes in their digestive tract, the gut microbiota. A wide range of factors influence microbiota regulation, including host characteristics, dietary patterns, and environmental and microbiological factors.

A dynamic equilibrium exists between the microbiota and the host, in which the former plays a role both locally and remotely in fundamental physiological processes such as inflammation and immunity. The microbes that live in our gut are capable of producing metabolites that protect the host from pathogens, but they can also produce molecules that are detrimental to the host when the relationship between the host and gut microbes is disturbed (a state known as dysbiosis) (Alvarez-Mercado et al., 2023). A wide range of evidence has shown that the gut microbiota plays a dual role in maintaining the health of the host and in the development of diseases such as liver disease (Zheng et al., 2020).

“Liver disease” refers to a variety of conditions that impair the ability of the liver to function normally. The progression of liver disease may result in scarring and more serious complications in the future. Nowadays, liver disease is a global burden that causes a tremendous socioeconomic cost (Giuffre et al., 2020). Advanced liver damage can manifest itself in the form of steatosis, fibrosis, and cirrhosis. These lesions can have alcoholic, viral, genetic, and metabolic origins. In fact, the increasing incidence of obesity and diabetes is causing non-alcoholic steatohepatitis to keep rising (Levičar et al., 2007).

The liver is intimately linked to the gut *via* the portal vein (a connection referred to as the gut-liver axis). Patients with cirrhosis develop gut dysbiosis, small bowel bacterial overgrowth, and increased gut wall permeability, favoring bacterial translocation and uptake of endotoxin which, in turn, induce hepatic and systemic inflammation (Arab et al., 2018).

Signals generated by diet, genetics, and the environment are integrated and communicate bidirectionally between the liver and the gut microbiota. During this reciprocal interaction, gut-derived products are transported directly to the liver *via* the portal vein. The intestinal mucosal and vascular barrier is the functional and anatomical structure that serves as a playground for the interactions between the gut and the liver, preventing microbes and toxins from dispersing throughout the body, while allowing nutrients to enter the circulation and be absorbed by the liver. In order to maintain the balance of the gut-liver axis, microbial communities need to be controlled, and the liver plays an important role in shaping intestinal microbial communities as part of this bidirectional communication. A number of interrelated levels of the gut-liver axis are disturbed by alcohol, including the gut microbiome, mucus barrier, epithelial barrier, and the production of antimicrobial peptides, which lead to an increase in microbial exposure and a proinflammatory environment within the liver (Albillos et al., 2020). This special issue contains several articles that provide an overview of the interaction between the gut microbiota and the immune system in liver surgery and liver disease.

## Interplay between gut microbiota and the immune system in liver surgery and liver diseases

This special issue of Frontiers in Cellular and Infection Microbiology includes five articles summarizing recent advances in the study of gut microbiota and the immune system in liver diseases.

Several characteristics of the gut microbiota have been studied in mice with nonalcoholic fatty liver disease (NAFLD), but it was unclear how the gut microbiota affects the abundance of intestinal metabolites. A high-fat diet was used to establish a mouse model of NAFLD in the experiment performed by Gu et al.. Their results showed a significant increase in the abundance of *Blautia*, unidentified-*Lachnospiraceae*, *Romboutsia*, *Faecalibaculum*, and *Ileibacterium*, while *Allobaculum* and *Enterorhabdus* decreased. Polyunsaturated fatty acids, vitamins, and nucleotides were significantly decreased, whereas amino acids, saturated fatty acids, and bile acids were increased. Based on correlation analysis and Model-based Integration of Metabolite Observations and Species Abundances analysis version 2.0, it appears that the gut microbiota did not affect the changes in lipids and bile acids. Still, it can reduce thiamine, pyridoxine, and promote L-phenylalanine and tyramine production (Gu et al.).

On the other hand, using quantitative real-time PCR, the microbiota from the feces of 181 obese individuals was compared. The authors compared gut microbiota of patients with obesity and NAFLD *versus* obese patients without NAFLD. They found a similar gut-dominant microbiota between groups. The number of *Faecalibacterium prausnitzii* colonies in the NAFLD group was, however, much lower than that in the simple obesity group. In both groups, *Bacteroides* were present in more than 65% of the cases. *Bacteroides*, *Clostridium leptum*, and *Clostridium butyricum* were present in feces samples of more than 80% of the cases of obesity with NAFLD, whereas *Bacteroides*, *C. butyricum*, and *F. prausnitzii* were present in more than 80% of the cases of simple obesity (Jin and Xu). Also, obesity-related NAFLD was investigated in order to identify potential contributory and preventive factors. The results of a multi-factor logistic regression analysis suggest that lymphocytes are associated with obesity with NAFLD, while *F. Prausnitzii* may act as a protective factor. Furthermore, the presence of *Bacteroides*, *C. leptum*, *C. butyricum*, and *Eubacterium rectale* positively impacts *F. prausnitzii*, while *Enterobacteriaceae* adversely impacts it (Jin and Xu).

In another study, an analysis of pleural effusion and ascites samples from 73 patients with end-stage liver disease using metagenomic next-generation sequencing identified 96 pathogens, including 47 bacteria (two *Mycobacterium tuberculosis*), 32 viruses, 14 fungi, and one parasite. Compared to conventional culture methods, metagenomic next-generation sequencing identified positive correlations between pathogens and disease in 42.5% of cases, a significant increase over conventional culture methods. The coincidence between metagenomic next-generation sequencing results and the final clinical diagnosis was 78.6% and 44.4%, respectively, for patients with confirmed positive bacteria detection and suspected positive bacteria detection. Moreover, the coincidence rate between metagenomic next-generation sequencing and confirmed fungal detection was 66.7%. It is noteworthy that five patients with fungal detection had alcoholic liver disease, accounting for 45.5% of all the patients with alcoholic liver disease. Furthermore, 32 strains of viruses were detected using metagenomic next-generation sequencing, primarily cytomegalovirus (62.5%) (Chen et al.).

Patients with primary liver cancer had higher levels of *Firmicutes* and *Actinobacteria* in their tongue coatings as compared to healthy individuals. In tongue coatings of healthy controls, *Fusobacteria*, *SR1_Absconditabacteria*_, and *Spirochaete* were more abundant than samples from patients with primary liver cancer. Additionally, thick or greasy tongue-coating patients had a higher abundance of *Fusobacteria* and *Actinobacteria* as compared to non-thick or greasy tongue-coating patients. Genomic pathways associated with replication, recombination, and protein repair were mainly enriched in tongue-coating patients with primary liver cancer. The tongue-coating microbiome signature includes 15 variables on a genus level to distinguish healthy controls from primary liver cancer. During training and validation, the signature performed well in terms of prediction accuracy (Zhang et al.).

Hepatitis, cirrhosis, and liver cancer constitute an important and common component of liver disease. Liver diseases are generally caused by inflammation and other pathogenic factors, resulting in the activation of hepatic stellate cells and excessive deposition of the extracellular matrix. It is important to note that chronic hepatitis patients are at risk of developing liver fibrosis, cirrhosis, and even life-threatening liver cancer, which pose a tremendous threat to public health. In their review article, Lui and Yang, 2023 report that liver microbiota and its metabolites have profound effects on the liver, as it is the first organ to come into contact with blood from the gut. This is due to leaky gut syndrome, an imbalanced microbiota being the trigger of the liver’s pathological response (Liu and Yang).

In summary, the five contributions to this special issue provide novel insights into the interaction between the gut microbiota and the immune system in liver diseases as well as into the existence of specific microbiota signatures for different types of diseases, and how these signatures might be modulated in order to improve the health of patients with liver diseases. It would be beneficial to investigate strategies for maintaining a balanced microbiota in liver disease patients (dietary interventions, microbe administrations, or fecal microbiota transplantations) in order to achieve more personalized and precise health.

## Author contributions

All authors listed have made a substantial, direct, and intellectual contribution to the work and approved it for publication. 
